# Two-Step Mechanism of Cyclin B Degradation Initiated by Proteolytic Cleavage with the 26 S Proteasome in Fish

**DOI:** 10.1038/s41598-020-65009-w

**Published:** 2020-06-02

**Authors:** Toshinobu Tokumoto, Md. Forhad Hossain, Md. Maisum Sarwar Jyoti, Md. Hasan Ali, Md. Babul Hossain, Mrityunjoy Acharjee, Md. Rezanujjaman, Mika Tokumoto

**Affiliations:** 1 Integrated Bioscience Section, Graduate School of Science and Technology, National University Corporation Shizuoka University, Ohya 836, Suruga-ku, Shizuoka 422-8529 Japan; 2 Biological Science Course, Graduate School of Science, National University Corporation, Shizuoka University, Oya 836, Suruga-ku, Shizuoka 422-8529 Japan; 3 Department of Bioscience, Faculty of Science, Shizuoka University, Shizuoka, 422 Japan; 4 CREST Research Project, Japan Science and Technology Corporation, Shizuoka, Japan

**Keywords:** Proteases, Proteasome

## Abstract

To complete meiosis II, cyclin B is degraded in a short period by the inactivation of M-phase promoting factor (MPF). Previously, we showed that the destruction of cyclin B was initiated by the ubiquitin-independent proteolytic activity of the 26 S proteasome through an initial cut in the N-terminus of cyclin (at K57 in the case of goldfish cyclin B). We hypothesized that this cut allows cyclin to be ubiquitinated for further destruction by the ubiquitin-dependent proteolytic pathway, which leads to MPF inactivation. In this study, we aimed to identify the ubiquitination site for further degradation. The destruction of cyclin B point mutants in which lysine residues in a lysine-rich stretch following the cut site of cyclin B had been mutated was analyzed. All the lysine point mutants except K57R (a point mutant in which K57 was substituted with arginine) were susceptible to proteolytic cleavage by the 26 S proteasome. However, the degradation of the K77R and K7677R mutants in *Xenopus* egg extracts was significantly slower than the degradation of other mutants, and a 42 kDa truncated form of cyclin B was detected during the onset of the degradation of these mutants. The truncated form of recombinant cyclin B, an N-terminal truncated cyclin BΔ57 produced as cut by the 26 S proteasome, was not further cleaved by the 26 S proteasome but rather degraded in *Xenopus* egg extracts. The injection of the K57R, K77R and K7677R cyclin B proteins stopped cleavage in *Xenopus* embryos. From the results of a series of experiments, we concluded that cyclin B degradation involves a two-step mechanism initiated by initial ubiquitin-independent cleavage by the 26 S proteasome at lysine 57 followed by its ubiquitin-dependent destruction by the 26 S proteasome following ubiquitination at lysine 77.

## Introduction

Proteolysis by the 26 S proteasome is usually attributed to a ubiquitin-dependent proteolytic pathway by adding ubiquitin protein to a substrate protein. However, many examples of degradation by a ubiquitin-independent proteolytic pathway that does not require addition of ubiquitin to substrate proteins^1^ such as p21, ornithin decarboxylase (ODC), p53, and retinoblastoma (Rb) proteins have been reported^2–6^. The ubiquitin-independent proteolytic pathway also has been discovered undergoes partial cleavage by the 26 S proteasome^7^, degradation by the 20 S proteasome^8–10^, which is the active center of the 26 S proteasome, and after monoubiquitination^11,12^, which differs from normal ubiquitin-dependent proteolysis in which a ubiquitin chain is added then degradation happen. Thus it is becoming clear that there are various modes of proteolysis by the proteasomes depending on the substrate. Recently, ubiquitin-independent proteolysis has been shown to play an important role in germ cell formation^13–15^. It was shown in *Xenopus* systems that the degradation of Dnd1, identified as one of the responsible genes of mouse teratoma, is due to ubiquitin-independent proteolysis^16^. It has long been known that proteasomes are present in large amounts in oocytes, it is of great interest that the degradation of key factors involved in the control of germ cell formation is due to ubiquitin-independent proteolysis. We have previously shown that cyclin B, a regulatory subunit of maturation or M-phase promoting factor (MPF), undergoes limited degradation at its N-terminus, and that this is the first reaction of cyclin B degradation^7^. The degradation of cyclin B is required for the transition from metaphase to anaphase^17^. Using biologically active recombinant goldfish cyclin B and purified 26 S proteasome allowed the study of cyclin degradation *in vitro*^7^. The 26 S proteasome cleaved recombinant 49 kDa cyclin B at lysine 57 (K57), producing a 42 kDa truncated form. The 42 kDa cyclin was also produced by the proteolytic cleavage of native cyclin B, forming MPF complex with cdc2, and a fragment transiently appeared during egg activation which cause rapid cyclin degradation *in vivo*. A point mutant cyclin B at cutting site of the 26 S proteasome (K57R) was resistant to both proteolytic cleavage by the 26 S proteasome and degradation in *Xenopus* egg extracts. The results suggested that the degradation of cyclin B was initiated by ubiquitin-independent proteolytic activity of the 26 S proteasome through an initial cut in the N-terminus of cyclin. We also hypothesize that this cut allowed the cyclin to be ubiquitinated for its further destruction by the ubiquitin-dependent activity of the 26 S proteasome, leading to MPF inactivation.

In this study, further experiments were conducted to show the molecular mechanism of cyclin degradation, especially the identification of the lysine residue that is destined to be ubiquitinated. Here, we propose a two-step mechanism of fish cyclin B degradation mediated by the ubiquitin-independent and ubiquitin-dependent proteolytic activity of the 26 S proteasome.

## Results

### Restricted proteolytic cleavage of cyclin B mutants by the 26 S proteasome

**Figure 1:**
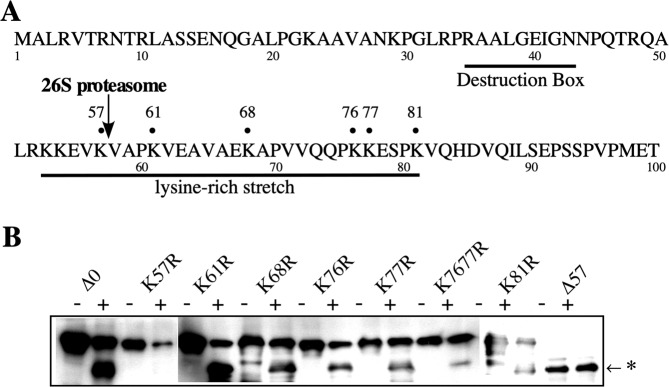
Proteolytic cleavage of *E. coli*-produced goldfish cyclin Bs by the 26 S proteasome purified from immature goldfish oocytes. (**A**) Amino acid sequence of the N-terminal region of goldfish cyclin B. The site cleaved by the 26 S proteasome (C-terminus of K57) and sites mutated from lysine to arginine residues are indicated by dots. The destruction box and lysine-rich stretch are also indicated. (**B**) Proteolytic cleavage of cyclin Bs by purified 26 S proteasome. Cyclins (5 μg/ml) were incubated for 60 min at room temperature with (+) or without (−) purified 26 S proteasome (60 μg/ml) in reaction buffer (100 mM Tris-HCl, 5 mM MgCl_2_, 0.04 mM ATP, pH 7.6). The samples were combined with Laemmli’s SDS sample buffer and separated by SDS-PAGE. Cyclin B was detected by immunoblotting against an anti-goldfish cyclin B (B63) monoclonal antibody. The position to which the cleaved cyclin B migrated is indicated by an asterisk.

To identify the ubiquitination site of goldfish cyclin B, cyclin B point mutants of lysine residues in a lysine-rich stretch following the 26 S proteasome cut site were produced (K61R, K68R, K76R, K77R, K81R; lysine was converted to arginine) (Fig. 1A). In the case of a lysine doublet, the double K7677R mutant was also produced. The 26 S proteasome showed peptidase activity and the activity against K-MCA was about 6.5 times higher than R-MCA hydrolyzing activity (Supplementary Fig. S1). Thus it is suggested that C-terminal of lysine residue is more susceptible for the 26 S proteasome than arginine residue. Thus we selected arginine for amino acid exchange. Before performing a destruction assay with cyclin B mutants, we addressed the susceptibility of the cyclin B mutants to proteolytic cleavage by the 26 S proteasome. As described previously, all the full-length goldfish cyclin B point mutants produced in *E. coli* (cyclin Δ0) except K57R, a mutant at the 26 S proteasome cut site, were good substrates for the 26 S proteasome. After the mutants were cut by the 26 S proteasome, 42 kDa cyclins were produced (Fig. 1B). The truncated form of cyclin B (cyclin Δ57) produced after being cut by the 26 S proteasome remained unchanged after incubation with the 26 S proteasome. These results indicated that the C-terminus of K57 is a cut site for the 26 S proteasome and that no further cutting is mediated by the direct cleavage of the 26 S proteasome. We then examined whether or not cyclin B from other species were cleaved by the 26 S proteasome, like goldfish cyclin B. Cyclin Bs, zebrafish cyclin B1, *Xenopus* cyclin B2 and Medaka cyclin B1, were cleaved by goldfish 26 S proteasome and produced intermediate as goldfish cyclin B (Supplementary Fig. S2). In previous study, we showed that *Xenopus* 26 S, but not 20 S proteasomes, cleaved the N-terminus of goldfish cyclin B and produced the 42 kDa intermediate as goldfish 26 S proteasome^7^. These results suggested that cyclin Bs were degraded by a similar mechanisms in these species.

### Destruction analysis in *Xenopus* egg extracts

We then examined the susceptibility of cyclin B mutants to degradation in a *Xenopus* cell-free system that contained everything necessary for cyclin degradation^17^.

**Figure 2:**
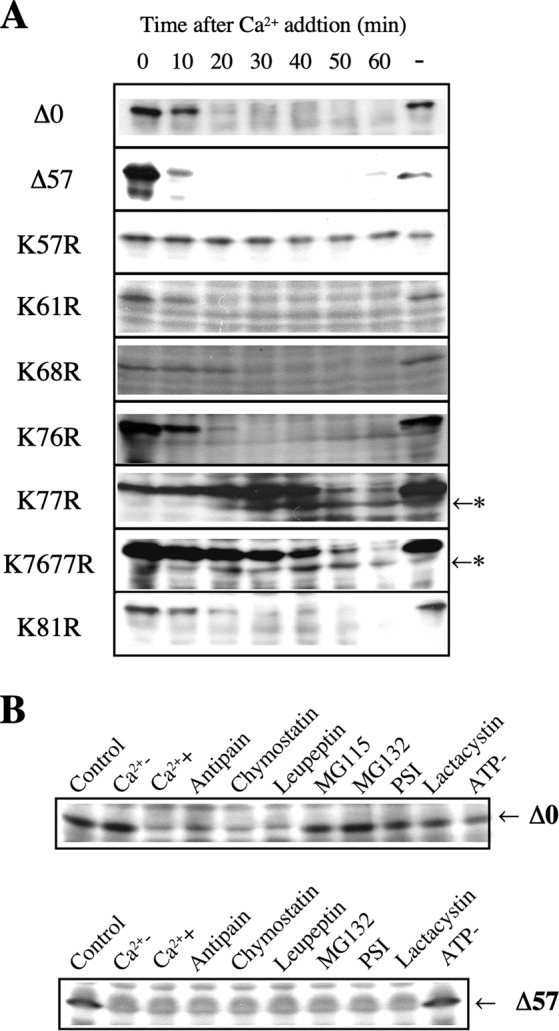
Degradation of goldfish cyclin Bs in *Xenopus* egg extracts. (**A**) *E. coli*-produced goldfish cyclins were added to a *Xenopus* egg extract at a final concentration of 5 μg/ml. The incubations proceeded in the absence (−Ca^2^ ^+^ ) or presence (+Ca^2^^ +^ ) of 0.4 mM CaCl_2_ for the indicated times. Cyclin degradation was terminated by transferring a portion of the reaction mixture into SDS sample buffer at the indicated times. Cyclin B was detected with the B63 antibody. The position of the cleaved cyclin B is indicated by an asterisk. (**B**) Effect of protease and proteasome inhibitors on cyclin B degradation in *Xenopus* egg extracts. *E. coli*- produced goldfish cyclin Δ0 or Δ57 was added to *Xenopus* egg extract at a final concentration of 5 μg/ml. Incubations proceeded after addition of (+Ca^2^^ + ^) of 0.4 mM CaCl_2_ in the presence of various inhibitors at 50 μM for 60 min except absence of (−Ca^2^^ + ^). The sample without incubation was included as Control. ATP- indicates the addition of ATP-depletion system (1 μg/ml hexokinase and 10 mM glucose). Cyclin B was detected by the B63 antibody.

**Figure 3:**
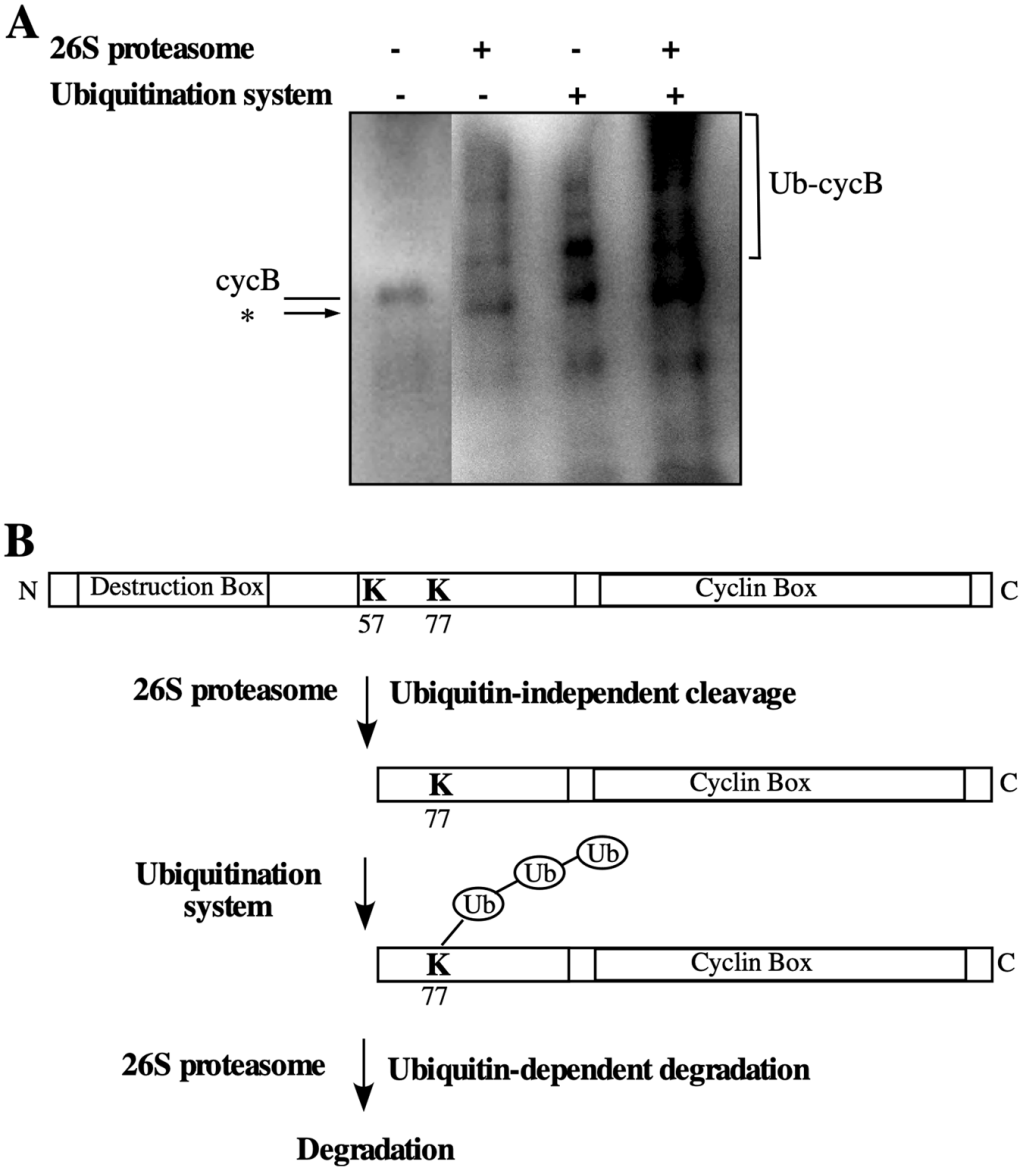
Ubiquitination of cyclin B after proteolytic cleavage by the 26 S proteasome. (**A**) Proteolytic cleavage and ubiquitination of native cyclin B. The MPF complex in mature goldfish oocytes was prepared using suc1-beads^35^. The beads were washed with buffer (50 mM Tris-HCl, 20% glycerol, 10 mM 2-mercaptoethanol, 0.1 mM ATP, pH 7.5) and shaken in the absence (−) or presence (+) of 60 μg/ml of the 26 S proteasome at room temperature with agitation. Samples were treated with SDS sample buffer (2 lanes at left side), or 26 S proteasomes were washed out with buffer (2 lanes at right side). Then the beads were shaken in the ubiquitination system (goldfish recombinant E1, E2-C, APC11 and ubiquitin) at room temperature with agitation (Ubiquitination system + ). Samples were treated with SDS sample buffer. All samples were immunoblotted against anti-goldfish cyclin B polyclonal antibodies^42^. The truncated cyclin B produced by the 26 S proteasome proteolytic cleavage is indicated by an asterisk. Protein bands of ubiquitinated cyclin B are indicated by a square bracket (Ub-cycB). (**B**) A model of the two-step degradation of cyclin B upon fish fertilization. Lysine residues that are target sites for proteolytic cleavage by the 26 S proteasome (K57) and ubiquitination (K77) are indicated. Ubiquitin is indicated as Ub.

As described previously, full-length goldfish cyclin B (cyclin Δ0) was completely degraded within 20–30 min of adding Ca^2+^ to *Xenopus* egg extracts, although it was stable in the absence of Ca^2^^ +^ (Fig. 2A). The K57R point mutant was not degraded in *Xenopus* extracts, as described (Fig. 2A)^7^. Point mutants in which a lysine residue was converted to arginine, K68R, K76R and K81R, were degraded similar to wild-type cyclin B. In contrast to these mutants, the degradation of the K77R and K7677R mutants was extremely slow, and a band corresponding to the 42-kDa cyclin fragment appeared during degradation in accordance with the disappearance of full-length cyclin B after the addition of Ca^2^ ^+^ (Fig. 2A). It is confirmed by co-electrophoresis that the size of the 42-kDa cyclin fragment from cyclin K77R appeared during degradation in *Xenopus* egg extract was same as intermediate produced by the proteolytic cleavage with the 26 S proteasome (Supplementary Fig. S3). Also it is confirmed that the size of 42-kDa cyclin fragment from K77R and K7677R mutants were same. These results suggested that these mutants were cleaved by the 26 S proteasome but were not ubiquitinated and remained undegraded. Consequently, this strongly suggests that K77 is a target site for ubiquitination. N-terminal truncated cyclin Δ57 was also degraded in *Xenopus* egg extracts (Fig. 2A). Protease inhibitors of microbial origin (antipain, chymostatin and leupeptin) did not block the cyclin degradation, but proteasome specific inhibitors (MG115, MG132, PSI and lactacystin)^18–21^, blocked the degradation of cyclin Δ0 (Fig. 2B). On the contrary, all the protease inhibitors including proteasome specific inhibitors did not show any inhibitory activity against degradation of cyclin Δ57. Cyclin Δ57 was degraded even without stimulation by Ca^2+^ addition. Only the depletion of ATP from the egg extract blocked the degradation of cyclin Δ57. The result suggested that ubiquitination system in *Xenopus* egg extract was constitutively active and cyclin Δ57 was ubiquitinated without activation by Ca^2+^ addition. These results suggested that initial cleavage at the appropriate position produces a susceptible site for ubiquitination and further degradation. In previous study, we showed that the 26 S proteasome cleave native cyclin B in a complex with cdc2. By using *in vitro* ubiquitination system from goldfish^22^, we addressed that truncated native cyclin B in a complex with cdc2 could be a substrate for ubiquitination or not. As shown in Fig. 3A, cyclin B was ubiquitinated in great magnitude after proteolytic cleavage by the 26 S proteasome. This result also supported the notion that initial proteolytic cleavage by the 26 S proteasome is a prerequisite before ubiquitination.

From a series of results, a two-step degradation mechanism of goldfish cyclin B initiated by ubiquitin-independent cutting at K57 followed by ubiquitination at K77 and the complete degradation of ubiquitinated cyclin B was proposed (Fig. 3B).

### Cell division arrest assay using 2-cell-stage *Xenopus* embryos

**Figure 4:**
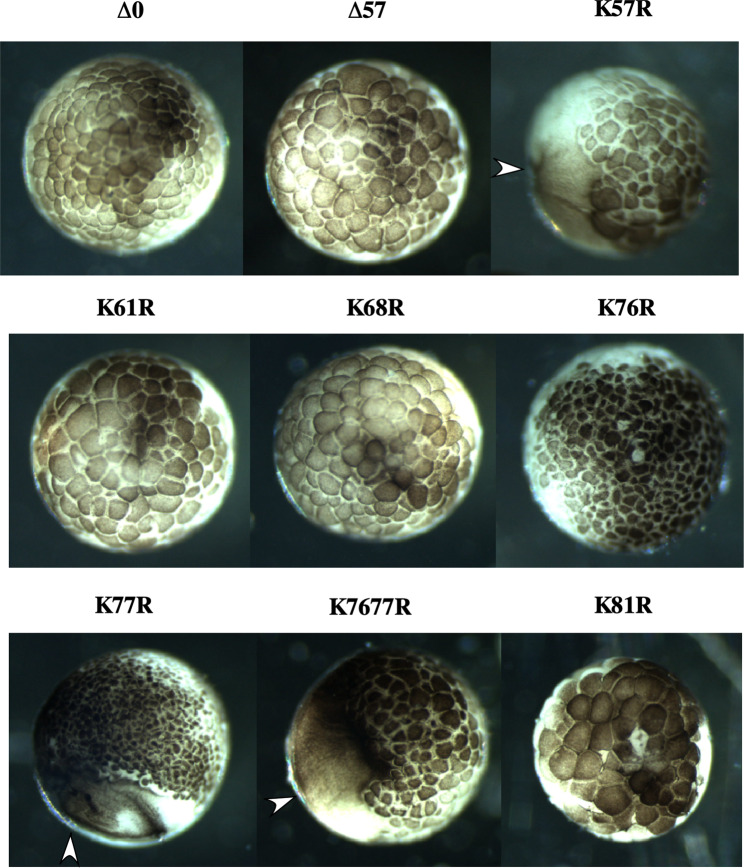
Cell division arrest assay using 2-cell-stage *Xenopus* embryos. Purified recombinant cyclin Bs at a final concentration of 5 μg/ml were microinjected into one side of 2-cell-stage *Xenopus* embryos. The embryos were incubated until stages 7–8 and photographed. The formation of a blastomere stopped the cleavage, is indicated by the arrowhead.

The activities of undegradable cyclin Bs were confirmed by a cell division arrest assay using 2-cell-stage *Xenopus* embryos. The above *in vitro* experiments showed that a mutant at the 26 S proteasome cut site and mutants at the possible ubiquitination site, K77R and K7677R, were resistant to degradation and inactivation. We further tested these results in living cells. In this assay, destruction-resistant cyclin B maintained the activity of M-phase promoting factor, and cells could not exit M phase. Then, the cell cycle was stopped. When we injected a series of cyclin Bs, only the Δ0K57R, K77R and K7677R cyclins stopped cleavage, as expected (Fig. 4). These results strongly supported the two-step mechanism of cyclin B degradation.

## Discussion

The results in this study suggested that degradation of cyclin B in fish is initiated by ubiquitin-independent limited cleavage by the 26 S proteasome, and complete degradation is achieved by ubiquitin-dependent proteolysis.

Previously, we proposed that the initial reaction of cyclin B degradation is the restricted cleavage of its N-terminal end by the 26 S proteasome^7^. Additionally, we suggested that the restricted cleavage of the N-terminus of cyclin B by the 26 S proteasome allows the cyclin to be ubiquitinated. Proteins marked for degradation by the ubiquitin pathway are ligated to ubiquitin through their lysine amino acid groups and then degraded by the 26 S proteasome^23^. Ubiquitination is carried out by the transfer of ubiquitin by three enzymes (E1, E2 and E3). Among the multiple species of E2s, a cyclin-selective ubiquitin-conjugating enzyme (UBC) family member, E2-C, was reported to ubiquitinate cyclin B in an anaphase-promoting factor (APC/cyclosome)-dependent manner^24^. The mRNA expression patterns of E2-C and cyclin B in goldfish showed their clear relationship^25^. The ubiquitination of cyclin BΔ57 was demonstrated by an *in vitro* experiment using ubiquitinating enzymes^22^. These findings suggest that cell cycle-specific cyclin degradation is mediated by a two-step mechanism by a ubiquitin-independent and ubiquitin-dependent proteolytic system. Then, we analyzed the ubiquitination sites involved in further degradation. A web-based program can be used to predict the ubiquitination sites of proteins (http://www.ubpred.org)^26^. With this program, the ubiquitination sites of goldfish cyclin B were predicted to be K68, K76, K77 and K81. Interestingly, these predicted sites are present at the remaining region N-terminal to the 26 S proteasome cut site. The N-terminal region of cyclin B is unfolded and does not contribute to binding with Cdk1 to form the MPF complex^27–29^. Thus, it is highly possible that the predicted lysine residues are target sites for ubiquitination. We next prepared point mutants of all the lysine residues in the lysine-rich stretch following the cut site of cyclin B. Then, we cleaved the point mutants with the 26 S proteasome and analyzed their destruction analysis in *Xenopus* egg extracts. All the lysine residue point mutants except K57 were susceptible to proteolytic cleavage by the 26 S proteasome. However, the degradation of the K77R and K7677R mutants in *Xenopus* egg extracts was significantly slower than that of the other mutants, and a 42 kDa truncated form of cyclin B was detected during the onset of the degradation of these mutants. The truncated form of recombinant cyclin B, N-terminal truncated cyclin BΔ57 produced after being cut by the 26 S proteasome, was not further cleaved by the 26 S proteasome but rather degraded in *Xenopus* egg extracts. From a series of experiments, we concluded that the mechanism of fish cyclin B degradation was initiated by an initial ubiquitin-independent cleavage by the 26 S proteasome at lysine 57, followed by ubiquitination at lysine 77 and then degraded ubiquitin-dependently by the 26 S proteasome (Fig. 3B).

We further demonstrated that the 26 S proteasome purified from immature oocytes can cleave cyclin B, but the 26 S proteasome purified from mature oocytes did not^30^. From these results, it is clear that the 26 S proteasome activities in these purified fractions are different.

These findings suggest that some inhibitory mechanisms against the proteasome itself prevent cyclin B degradation, at least during metaphase II arrest. Then, we hypothesize that the destruction is primarily controlled by the activity of the 26 S proteasome. To investigate the regulatory mechanism of the 26 S proteasome during the meiotic cell cycle, we compared the proteasomal components of these purified fractions. Then, we found that the presence of two protein bands, corresponding to molecular weights of 30 kDa and 62 kDa, differed between 26 S proteasomes from immature and mature oocytes^31^. The 30 kDa protein was the α4 subunit, which is one of the α-subunit groups of the 20 S proteasome, and the 62 kDa protein was a homologue of CCTε, a component of eukaryotic molecular chaperones. Phosphatase treatment of the 26 S proteasome revealed that a part of the α4 subunit of the goldfish 20 S proteasome, *α4_ca*, was phosphorylated in G2 phase and dephosphorylated in M phase. The kinase for the α4 subunit was identified as casein kinase Iα (CKIα)^32^. The α4 subunit was shown to be phosphorylated by CKIα in a meiotic cell cycle-dependent manner. These results suggest that the phosphorylation of the α4 subunit of the 26 S proteasome by CKIα might be involved in the regulation of the meiotic cell cycle.

Further studies should reveal how protein modifications regulate the activity of the 26 S proteasome during oocyte maturation and egg activation to control the cell cycle by regulating cyclin stability.

## Methods

### Animals

Goldfish were purchased from a local supplier and maintained at 15 °C until use. The 26 S proteasomes were purified from immature goldfish ovaries or ovulated eggs by conventional column chromatography, as previously described^33^. *Xenopus laevis* was obtained from a dealer and maintained until use. *Xenopus* CSF-arrested egg extracts were prepared by the method of Murray *et al*.^17^. All animal experiments were carried out with approval from the Institutional Ethics Committee of Shizuoka University, Japan (approval no. 29F-2); the guidelines set by this committee for the usage of animals were strictly followed.

### Electrophoresis and immunoblot analysis

Electrophoresis proceeded as described by Laemmli^34^ using 12.5% gels under denaturing conditions. Cyclin B degradation was assessed by immunoblotting against anti-goldfish cyclin B (B63) monoclonal antibody^35^. Immunocomplexes were visualized using an ECL detection kit (GE Healthcare).

### Production of recombinant cyclin Bs

Full-length (Δ0) and N-terminal truncated (Δ57) goldfish cyclin Bs were produced, as previously described^36–38^. Mutant cyclin Bs in which lysines 57, 61, 68, 76, 77 and 81 were replaced by arginines (cyclin BΔ0K57R, K61R, K68R, K76R, K77R, K7677R, K81R) were produced as follows. A cDNA clone encoding full-length goldfish cyclin B^36^ was mutated using a site-directed mutagenesis system (Mutan-K, Takara) following a strategy based on the method of Kunkel^39^ according to the manufacturer’s instructions. Double-strand mutated cDNA was prepared by T3 polymerase using single strand cDNA and the following oligonucleotides containing exchanged nucleotides at the mutation site and a restriction enzyme site, as previously described^38^. Mutant clones were screened by proteolytic cleavage with restriction enzymes and confirmed by sequencing.

Recombinant proteins were produced in *E. coli* BL21 (DE3) and purified by SDS-PAGE, followed by electroelution from the gel, as described previously^36^.

### Cell division arrest assay using 2-cell-stage *Xenopus* embryos

Fertilized *Xenopus laevis* eggs were prepared by *in vitro* fertilization, as previously described^40^. Purified recombinant cyclin Bs at a final concentration of 5 μg/ml were microinjected into one side of 2-cell-stage *Xenopus* embryos. The embryos were incubated until stages 7–8 and photographed. The cell cycle arrest activity of mutant cyclin Bs was assessed after the cleavage of the injected blastomere did or did not stop^41^.

## Supplementary information

Supplementary Figures.
