# Supplementary material for: Two-Step Mechanism of Cyclin B Degradation Initiated by Proteolytic Cleavage with the 26 S Proteasome in Fish
Source: Sci Rep. 2020 Jun 2;10:8924. doi: 10.1038/s41598-020-65009-w (PMC7265292; doi:10.1038/s41598-020-65009-w)

Supplementary Information for

**Two-Step Mechanism of Cyclin B Degradation Initiated by Proteolytic Cleavage with the 26S Proteasome in Fish.**

**.**

**Toshinobu Tokumoto^1,2^*, Md. Forhad Hossain^2^, Md. Maisum Sarwar Jyoti^2^, Md. Hasan Ali^1^, Md. Babul Hossain^3^, Mrityunjoy Acharjee^1^, Md. Rezanujjaman^1^, Mika Tokumoto^4^+**

^1^Integrated Bioscience Section, Graduate School of Science and Technology, National University Corporation Shizuoka University, Ohya 836, Suruga-ku, Shizuoka 422-8529, Japan. ^2^Biological Science Course, Graduate School of Science, National University Corporation, Shizuoka University, Oya 836, Suruga-ku, Shizuoka 422-8529, Japan

^3^Department of Bioscience, Faculty of Science, Shizuoka University, Shizuoka 422, Japan. ^4^CREST Research Project, Japan Science and Technology Corporation, Japan.

+Address at the time of this study.

* Correspondence and requests for materials should be addressed to T.T. ([tokumoto.toshinobu@shizuoka.ac.jp](mailto:tokumoto.toshinobu@shizuoka.ac.jp))

**Figure caption of Supplementary Figures**

**Supplementary Figure. S1 Peptidase activities of the 26S proteasome purified from immature goldfish oocytes.**

Peptidase activities of the 26S proteasome against fluorescent peptide (K-MCA, R-MCA and Suc-LLVY-MCA) were measured as previously described ^1^. Concentrations of fluorescent peptides were 0.1 mM for K-MCA and R-MCA, 0.01 mM for Suc-LLVY-MCA. Fluorescent intensity was measured by microplate reader at excitation wavelength 360 nm and emission at 460 nm. Data are average of quadruplicate and vertical bar indicates standard deviation.


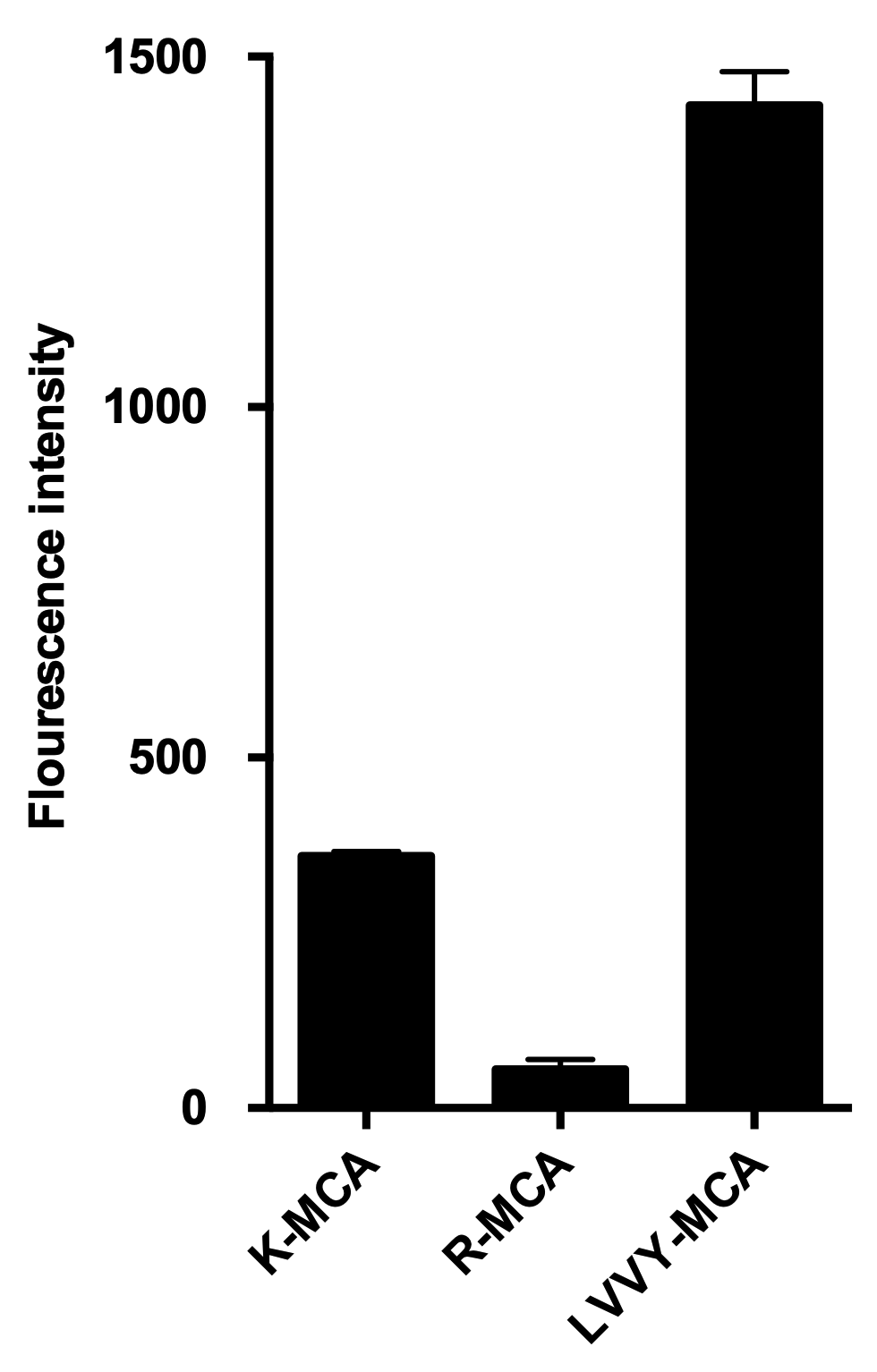


**References**

1 Tokumoto, T. & Ishikawa, K. A novel "active" form of proteasomes from *Xenopus laevis* ovary cytosol. *Biochem Biophys Res Commun* **192**, 1106-1114 (1993).

**Supplementary Figure. S2 Digestion of *E. coli*-produced zebrafish, *Xenopus* and Medaka cyclin Bs by the 26S proteasome purified from immature goldfish oocytes.**

Digestion of cyclin Bs by purified 26S proteasome. Cyclins (5 μg/ml) were incubated for 60 min at room temperature with (+) or without (-) purified 26S proteasome (60 μg/ml) in reaction buffer (100 mM Tris-HCl, 5 mM MgCl_2_, 0.04 mM ATP, pH 7.6). The samples were combined with Laemmli's SDS sample buffer and separated by SDS-PAGE. Cyclin B was detected by immunoblotting against an anti-His-tag polyclonal antibodies (MBL, Nagoya, Japan). The position to which the digested cyclin B migrated is indicated by an asterisk.

**
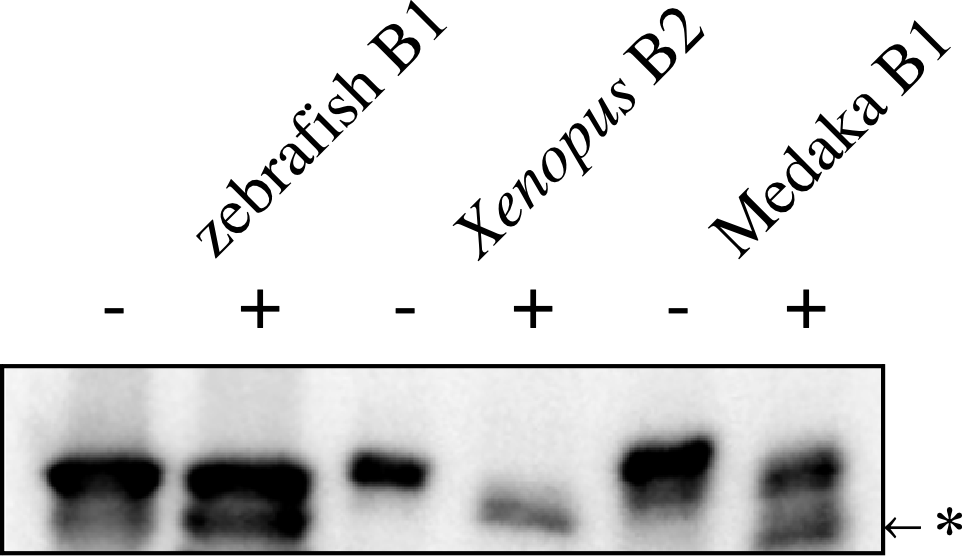
**

**Supplementary Figure. S3** **Co-electrophoresis of cyclin Bs digested by the 26S proteasome and intermediate products produced in *Xenopus* egg extracts.**

(A) Incubation mixtures of cyclin B K77R and K7677R in *Xenopus* extract for 30 min were resolved by SDS-PAGE. Cyclin B (cyc B) was detected by immunoblotting against an anti-goldfish cyclin B (B63) monoclonal antibody. The position to which the digested cyclin B migrated is indicated by an asterisk. (B) Reaction mixture of digested cyclin B K77R by purified 26S proteasome for 60 min and incubation mixture of cyclin B K77R in *Xenopus* extract for 30 min were combined (Mixture) or separately resolved by SDS-PAGE. Cyclin B was detected by immunoblotting against an anti-goldfish cyclin B (B63) monoclonal antibody. The position to which the digested cyclin B migrated is indicated by an asterisk.

Note: Cyclin B K77R protein migrated to different position after mixed with *Xenopus* egg extracts.


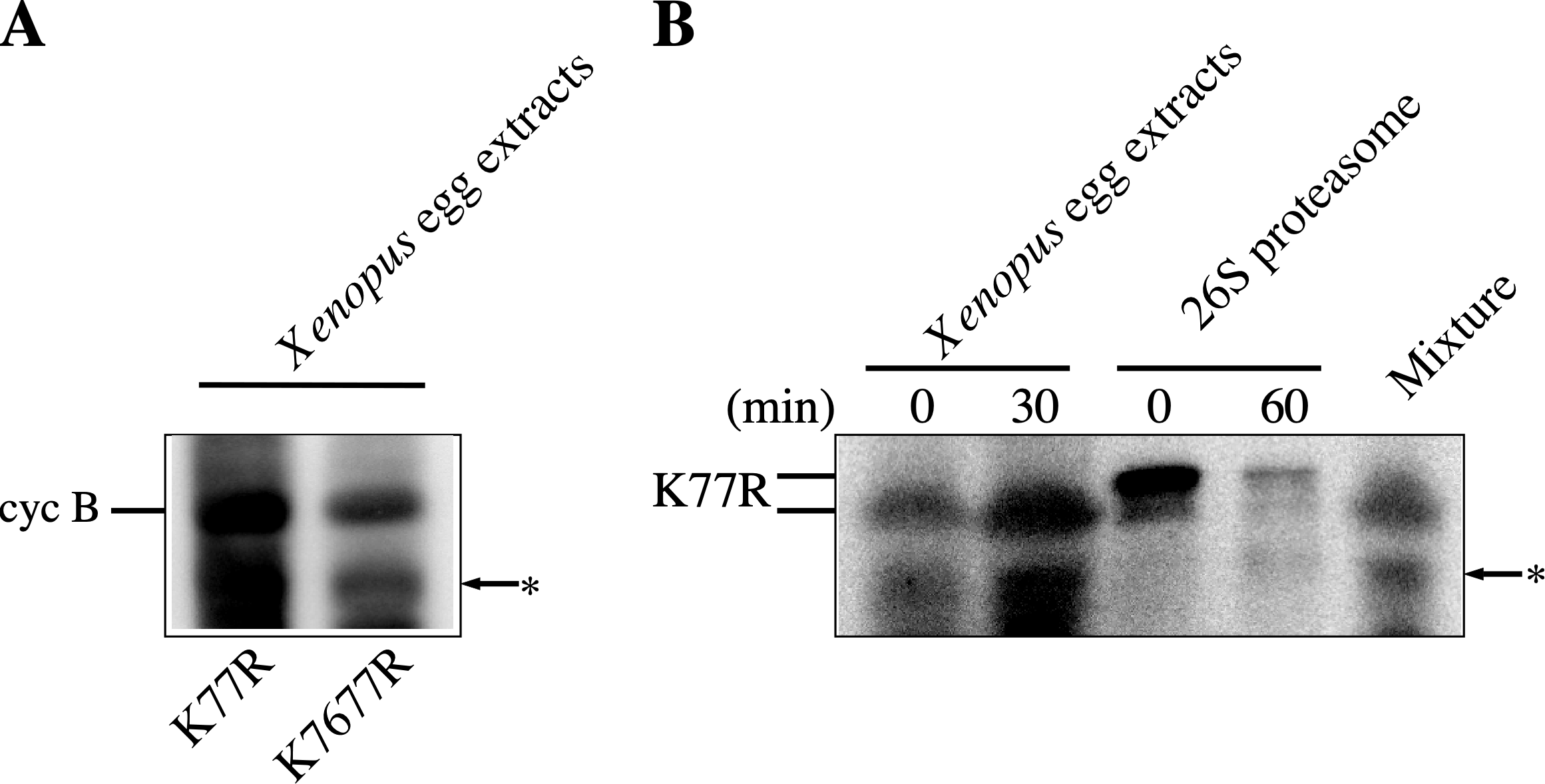

Supplement: Supplementary file 1 — Supplementary Figures. [file 41598_2020_65009_MOESM1_ESM.docx]
